# The unusual (*syn*-/*anti*-)_2_ conformation of a di­meth­oxy­pyrimidyl-based tennimide

**DOI:** 10.1107/S2056989023006837

**Published:** 2023-08-23

**Authors:** Pavle Mocilac, Fabian Pohl, John F. Gallagher

**Affiliations:** a School of Chemical Sciences, Dublin City University, Dublin 9, Ireland; b Fakultät für Chemie und Mineralogie, Universität Leipzig, Johannisallee 29, 04103 Leipzig, Sachsen, Germany; Universidade de Sâo Paulo, Brazil

**Keywords:** conformation, macrocycle, meth­oxy, pyridine, pyrimidine, tennimide, tetra­mer, crystal structure

## Abstract

The tennimide macrocycle, (**I**) (C_52_H_40_N_16_O_16_.0.167H_2_O), was synthesized from 2-amino-4,6-di­meth­oxy­pyrimidine and pyridine-2,6-dicarbonyl dichloride. Compound (**I**) represents the first tennimide incorporating pyridine rings in the macrocycle scaffold. In the macrocycle ring, the carbonyl groups at each successive dicarbon­yl(pyridine) moiety adopt the (*syn*/*anti*)_2_ conformation. This contrasts with all previously reported tetra­imide macrocycles, which exhibit the (*syn*)_4_ conformation. The effect is to close any potential cavity or *niche* by having two of the central pyridine C_5_N rings aligned close to each other [with closest pyridine *Cg*⋯*Cg* ring centroid separations of 3.5775 (19) Å; closest C⋯C = 3.467 (5) Å]. A partial occupancy water mol­ecule (with s.o.f. = 0.167), resides with its oxygen atom on a twofold axis at hydrogen-bonding distances to the carbonyl O atom, in a mol­ecular *niche* between two pyridine rings. Macrocyles of (**I**) have all six C=O groups and all eight meth­oxy O atoms present on the macrocycle surface. However, all twelve N atoms are effectively shielded on steric grounds from any potential inter­molecular inter­actions. The remaining two C=O O atoms inter­act with the partial occupancy water mol­ecule *via* two O—H⋯O=C hydrogen bonds. Macrocycles of (**I**) stack as one-dimensional chains along the *b*-axis direction with primary inter­molecular inter­actions involving weak C—H⋯O=C/OCH_3_/H_2_O contacts. Chains inter­lock weakly *via* meth­oxy–meth­oxy C—H⋯O inter­actions into two-dimensional sheets.

## Abstract 2

The tennimide macrocycle (**I**) resides on a twofold axis and adopts an alternate (*syn*/*anti*)_2_ conformation with no inner cavity (niche), unlike all previously reported (*syn*)_4_ tennimide conformations. A partial occupancy water mol­ecule (with 0.167 site occupancy) at hydrogen-bonding distances to two symmetry-related carbonyl O atoms (as O—H⋯O=C) resides on the twofold axis in a mol­ecular *niche* between two of the pyridine rings. Macrocycle aggregation occurs as one-dimensional chains along the (010) direction.

## 1. Chemical context

Developments in macrocyclic chemistry continue with an emphasis on structure, function and developing new architectures with pendant functional groups. An aim is to achieve new applications in coordination chemistry, nanoscience, natural products, medicinal chemistry and applied sciences (Böhmer, 1995 ▸; Vicens & Harrowfield, 2007 ▸). Macrocyclic science now spans several scientific fields and finds applications across a vast range of chemical, physical and biomedicinal sciences (Gloe, 2005 ▸; Davis & Higson, 2011 ▸).

Macrocycles usually contain donor atoms such as O, N, S and P and are utilized with a wide variety of aliphatic groups (*e.g*. in crown ethers) and/or aromatic rings (*e.g.* in calixarenes, porphyrins) (Böhmer, 1995 ▸; Gloe, 2005 ▸). An objective is that particular functional groups are incorporated onto a scaffold to accommodate a wide range of metals, their oxidation states and coordination chemistry geometries. This originates from the initial crown ethers, through macrocycles such as calix[*n*]arenes (Böhmer, 1995 ▸) to macromolecular macrocycles (Davis & Higson, 2011 ▸). Constituent functional groups now span a relatively large range of types as incorporated into many macrocycles in the form of amides, pyridines, imides *etc*. (Pappalardo *et al.*, 1992*a* ▸,*b* ▸; Böhmer, 1995 ▸; Vicens & Harrowfield, 2007 ▸). Recently, considerable effort has been made to incorporate biological moieties (*e.g*. peptide chains) for a range of applications including artificial ion channels and transport (Xin *et al.*, 2015 ▸; Legrand & Barboiu, 2013 ▸).

In terms of macrocyclic conformations, there are many examples where a macrocycle is isolated in a defined, stable geometry and subsequently shown to adopt alternate conformations. Usually these can be structurally characterized and the different conformations may or may not be inter­convertible in solution or even in the solid state. For example, the cone conformation of calix[4]arenes has been well described (Andreetti *et al.*, 1979 ▸; Gutsche, 1983 ▸). In addition, the partial cone, *syn*-distal and *syn*-proximal conformations have been studied in many different calix[4]arene derivatives (Gutsche *et al.*, 1983 ▸; Ferguson *et al.*, 1992 ▸, 1993 ▸; Pappalardo *et al.*, 1992*a* ▸,*b* ▸; Shinkai, 1993 ▸). Even in tennimide chemistry, we were fortunate to isolate a tennimide known as (26IO)_4_ with three distinct solid-state geometries (Mocilac & Gallagher, 2013 ▸), though these are inter­convertible in solution. The three (26IO)_4_ conformations differ in terms of the size and apertures of the mol­ecular cavity. For the smaller trezimide (trimer) systems, two conformations have been isolated as the distinct ***P*** and ***R*** conformations (Mocilac & Gallagher, 2013 ▸). Therefore, isolation and characterization of a new macrocyclic conformation in a class of imide-based macrocycles is of inter­est to researchers studying imide-based and related macrocycles. Furthermore, researchers have continued to advance conformational analysis and especially with respect to non-rigid macrocycles (Bohle & Grimme, 2022 ▸). Their study utilized the automated generation of macrocyclic conformers using computational methods as applied to ^13^C-NMR data of flexible cyclo­alkanes. With such developments, new macrocyclic conformations will be postulated for a range of macrocycle types in tandem with synthetic experimental studies. In addition, it has recently been shown using synthetic strategies, the engineering of mol­ecular topology in pseudopeptidic macrocycles (Sharma *et al.*, 2017 ▸).

Of particular note is that Balakrishna and co-workers have studied phospho­rus-based systems using >PCH_3_ and >PC_6_H_5_ as linker groups between the isophthaloyl moieties (Balakrishna, 2018 ▸; Kashid *et al.*, 2017 ▸). This contrasts with the >N(pyridine) and >N(pyrimidine) linkers that we have studied to date. Structural examples of the P-based crystal structures as VAWVIB and VAWVOH are available on the CSD (Groom *et al.*, 2016 ▸). In addition, the highly constrained butterfly structures as ‘dimers’ have been reported as RAYFII and ZAFJAV (Saunders *et al.*, 2012 ▸; Pearce & Crossley, 2020 ▸). He and co-workers in their structures NUKZIF, NULHOU, NULHUA (Wang *et al.*, 2020 ▸) noted that such di­imides and polyimides are still relatively rare (Wang *et al.*, 2020 ▸). These systems are more closely related to the well-explored planar di­imides such as napththalene di­imide (Takenaka, 2021 ▸).

**Figure 1:**
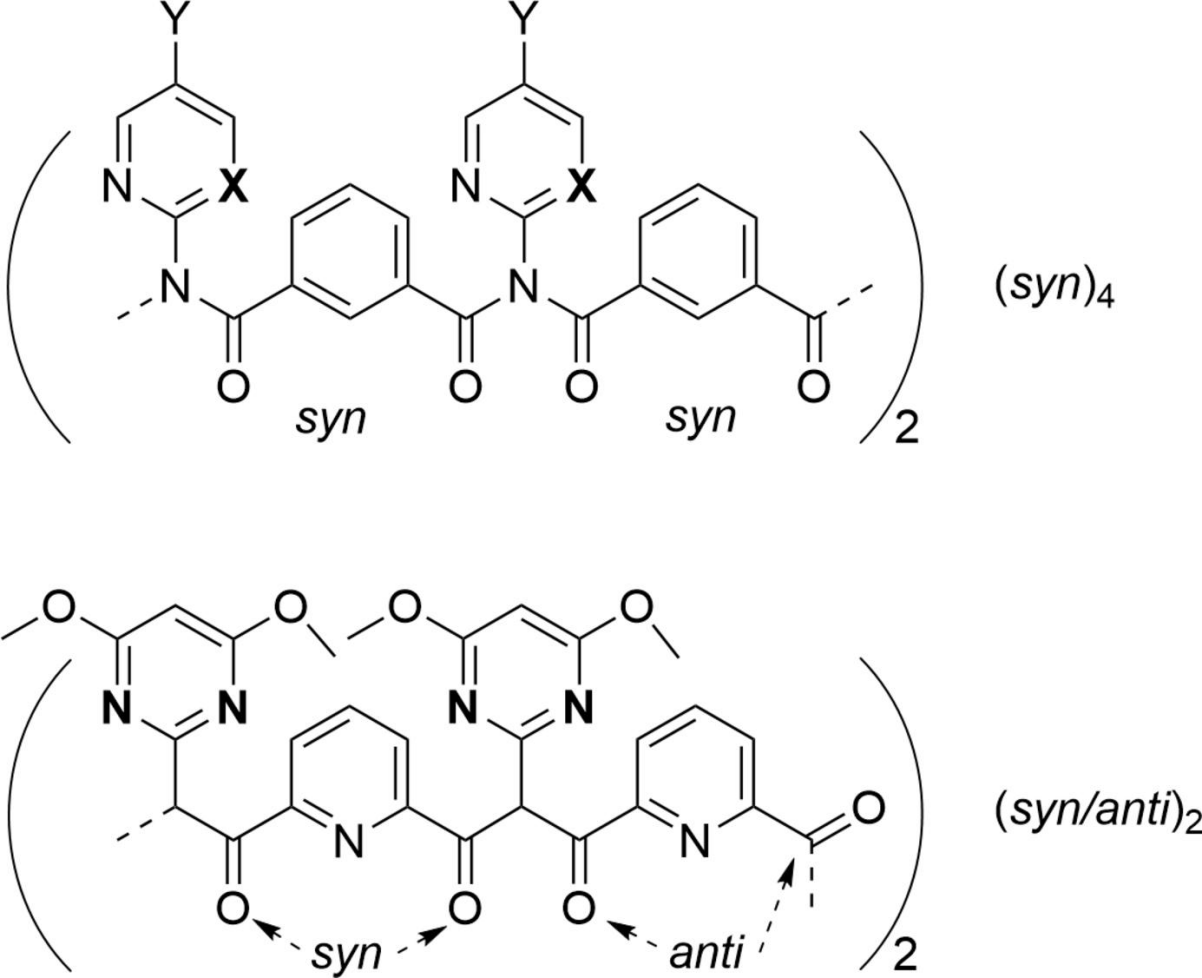
Schematic diagrams of (top) the tennimide (**I**) with the relative geometries depicted to minimize atom/group overlap and (bottom) the relative differences between the (*syn*)_4_ and (*syn*/*anti*-)_2_ conformations. The X and Y labels refer to: X = C or N and Y = H, CH_3_ or halogen atom (F, Cl, Br).

**Figure 2:**
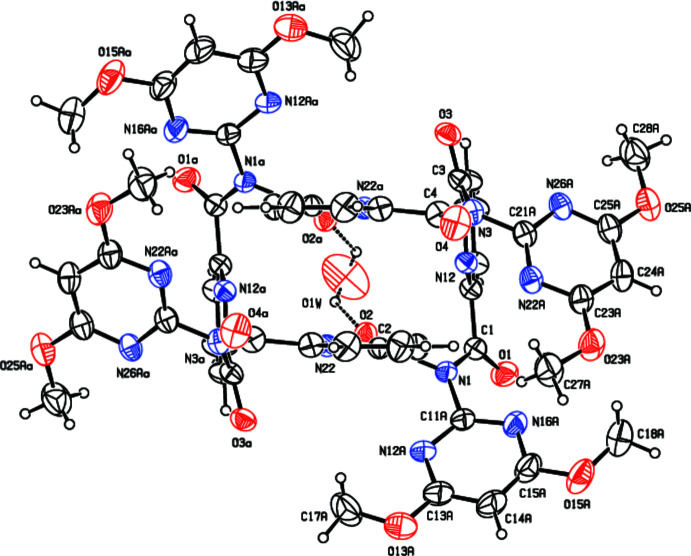
A view of (**I**) with the atomic-numbering scheme. Displacement ellipsoids are drawn at the 30% probability level for clarity. The partial occupancy water mol­ecule O1*W* is depicted with dashed lines representing the hydrogen bonding to (**I**). Symmetry code: (a) −*x*, *y*, −*z* + 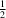 .

From previous benzamide studies (Donnelly *et al.*, 2008 ▸), we reported macrocyclic trimers (trezimides) and tetra­mers (tennimides) based on the isophthaloyl residue and imide linker group (Evans & Gale, 2004 ▸; Mocilac & Gallagher, 2013 ▸, 2014 ▸, 2016 ▸; Gallagher & Mocilac, 2021 ▸). These macrocycles with pendant pyridine, pyrimidine and pyridinyl ester groups are attached to the central scaffold and have potential to bind to metal complexes. Trimers and tetra­mers are typically synthesized and isolated in modest yields, together with oligomers and polymers from which they have to be carefully separated by column chromatography. We herein report a new pyridine-based tennimide macrocycle derived from 2-amino-4,6-di­meth­oxy­pyrimidine and pyridine-2,6-di­carbonyl­dichloride (Fig. 1 ▸) with an unusual (*syn*/*anti*)_2_ tennimide conformation (Fig. 2 ▸). The isolation of a new conformation using a pyridine-based scaffold demonstrates that these macrocycles can be investigated to exploit this new mol­ecular conformation. 
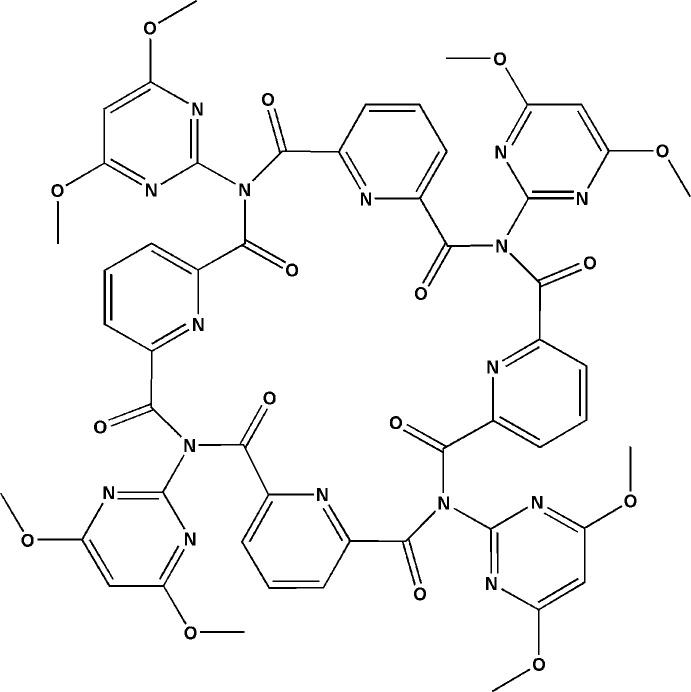


## 2. Structural commentary

The macrocycle (**I**), as synthesized from the condensation reaction of 2-amino-4,6-di­meth­oxy­pyrimidine with pyridine-2,6-di­carbonyl­dichloride, adopts a different (*syn*/*anti*)_2_ conformation to the (*syn*)_4_ seen in all reported tennimides (Evans & Gale, 2004 ▸; Mocilac & Gallagher, 2013 ▸, 2014 ▸, 2016 ▸) (Figs. 1 ▸, 2 ▸, 3 ▸). (**I**) represents the first tennimide synthesized using pyridine-2,6-dicarbonyl dichloride (in comparison to benzene di­carbonyl­dichloride) and thus has four pyridines incorporated into the scaffold. It comprises four pyridine N donor, eight pyrimidine N, eight carbonyl O donor atoms, together with eight meth­oxy groups and four aliphatic imide N atoms. However, the orientation of most aromatic N donors is not suitable for coordination due to shielding from the eight meth­oxy groups on the external surface of the macrocycle. There is no inner macrocyclic cavity because of the spatial arrangement of the pyridine rings in the (*syn*/*anti*)_2_ conformation. However, a partial occupancy water mol­ecule is observed in a macrocyclic niche at hydrogen-bonding distances to two imide carbonyl oxygen atoms (Fig. 2 ▸).

In all tennimides (Evans & Gale, 2004 ▸; Mocilac & Gallagher, 2013 ▸, 2014 ▸, 2016 ▸; Gallagher & Mocilac, 2021 ▸) the carbonyl group conformations with respect to the isophthaloyl residues is noted as *syn-*. Therefore, these macrocycles are classed as having a (*syn*)_4_ conformation (Mocilac & Gallagher, 2013 ▸, 2014 ▸, 2016 ▸; Gallagher & Mocilac, 2021 ▸). In (**I**), the pyridine conformation with respect to the carbonyl groups alternates as *syn*- and *anti-* and is defined as (*syn*/*anti*)_2_. This (*syn*/*anti*)_2_ conformation has no inter­nal cavity available to incorporate an ion or small mol­ecule (although a small niche is present). The geometric details are: two of the pyridine C_5_N rings are positioned close to each other [with closest pyridine ring centroid *Cg*⋯*Cg* separations = 3.5775 (19) Å; with closest C24⋯C24*a* = 3.467 (5) Å across the twofold axis; where *a* = −*x*, *y*, 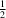 −  z]. In the macrocycle, the imide ‘(O=C)N’ hinge O=C⋯C=O torsion angles are oriented at −92.5 (4) and −91.59 (5)° for O1=C1⋯C2=O2 and O3=C3⋯C4=O4, respectively, which are typical of imide conformations in tennimides. However, the pyridine dicarbonyl moiety torsion angles differ with 87.0 (5)° for the *syn*-conformation (in O1=C1⋯C3=O3) and −144.89 (5)° for the *anti*-conformation (in O2=C2⋯C4=O4). The *syn-* torsion data is close to the average isophthaloyl (O=C)C_6_H_4_(C=O) torsion angle noted previously (Evans & Gale, 2004 ▸; Mocilac & Gallagher, 2013 ▸, 2014 ▸, 2016 ▸; Gallagher & Mocilac, 2021 ▸). In the four di­meth­oxy­pyrimidine rings, the meth­oxy groups all adopt similar conformations, as noted previously (Gallagher *et al.*, 2001 ▸). The proximally related pyridine rings are almost orthogonal to one another at 87.00 (10), 87.09 (10)° and with the opposite (distal-related) pyridine rings almost parallel at 9.25 (11), 0.97 (11)°. For symmetry-related pyridine rings, the C=O groups are positioned *anti*- with respect to one another.

A partial occupancy water mol­ecule O1*W* (site occupancy of 0.167) occupies a niche between two distal pyridines (with *syn-*related C=O groups), separated by *ca* 6 Å. The water mol­ecule O1*W* forms O—H⋯O=C hydrogen bonds [O⋯O = 2.97 (3) Å] with two symmetry-related carbonyl O2 atoms and two weaker C—H⋯O complete the hydrogen bonding (H⋯O = 2.49 Å).

## 3. Supra­molecular features

**Figure 4:**
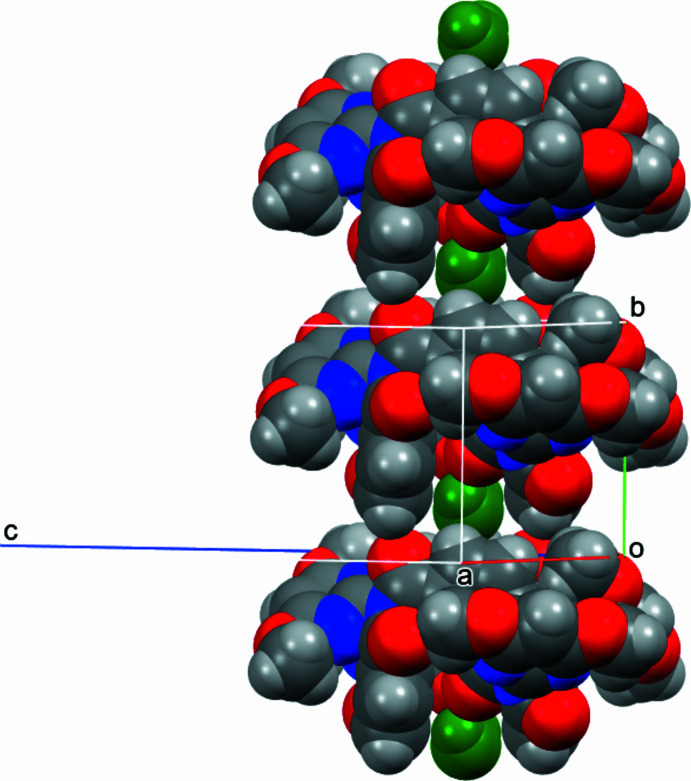
A view of the primary stacking in the crystal structure of (**I**) along the *b*-axis direction with atoms drawn as their van der Waals spheres and partial occupancy water O1*W* in green.

The primary inter­actions involving the macrocycle are a range of rather weak aliphatic/aromatic C—H⋯O contacts (Table 1 ▸) in the absence of strong hydrogen-bond donors. This arises in a system with a vast excess of potentially strong acceptor groups on the tennimide surface. Mol­ecules of (**I**) aggregate as 1D chains along the *b*-axis direction with primary inter­molecular inter­actions involving weak C—H⋯O=C/OCH_3_/H_2_O contacts (Fig. 4 ▸). Chains inter­lock weakly *via* meth­oxy⋯meth­oxy C—H⋯O inter­actions into 2D sheets. This type of 1D aggregation is quite common for this class of tennimide macrocycle. It was noted in the 1D macrocyclic stacking driven by C—Br⋯O=C/N_pyridine_ halogen bonding between mol­ecules in brominated tennimides (Mocilac & Gallagher, 2013 ▸).

## 4. Synthesis and crystallization

**Synthetic reaction conditions:**

Pyridine-2,6-dicarbonyl dichloride (2.041 g, 10 mmol, 1 eq.) was dissolved in 100 ml of dry CH_2_Cl_2_ (DCM) and a catalytic qu­antity of 14.4 mg 4-di­methyl­amino­pyridine (DMAP) with 4 ml (29.4 mmol, 3 eq.) of Et_3_N were added to the solution under N_2_. This solution was cooled to 255 K and 1.64 g (10 mmol, 1 eq.) of 4,6-di­meth­oxy­pyrimidin-2-amine dissolved in 40 ml dry of DCM were added. The solution was stirred for 72 h and thin layer chromatography (TLC: CH_2_Cl_2_/ethyl acetate, 4:1) indicated that conversion was incomplete (the related amino­pyridine reactants show full conversion within 24 h). The solution was then diluted to 250 ml with technical grade CH_2_Cl_2_, washed four times with 100 ml of NH_4_Cl solution (pH 4), dried using MgSO_4_ and the solvent mixture removed at reduced pressure. Filtration through silica gel (CH_2_Cl_2_/ethyl acetate, 2:1) was performed before final purification was attempted by column chromatography. The expected products were the acyclic (2:1), (3:2) and (4:3) mixed imide benzamides as well as longer chain oligomers and some polymeric materials. TLC indicated four bands (one of which was the pyrimidin-2-amine starting material) in addition to polymeric material at the bottom of the TLC plate. The polymer was mostly removed by preliminary filtration. Further attempts at purification again involved multiple steps of column chromatography. The first column (using *n*-hexa­ne/ethyl acetate, 2:1) resulted in poor separation and merely eliminated the remaining starting material. A subsequent column (CH_2_Cl_2_/ethyl acetate, 10:1) gave some separation and two products were isolated. The first product is the (2:1) pyridine-2,6-dicarboxamide and the second was shown to be a (4:4) macrocyclic tennimide (**I**) isolated in a relatively low yield of *ca* 5%. The final major product could not be isolated and purified.

***N*** **^2^**, ***N*** **^6^-bis­(4,6-di­meth­oxy­pyrimidin-2-yl)pyridine-2,6-dicarb­oxamide (2:1 product) ^1^H-NMR** (400 MHz, CDCl_3_): δ = 10.24 (*s*, 2H, N**H**), 8.50 (*d*, *J* = 7.8 Hz, 2H, Ar—**H**), 8.12 (*t*, *J* = 7.8 Hz, 1H, Ar—**H**), 5.81 (*s*, 2H, Ar—**H**), 3.94 (*s*, 2H, O—C**H_3_**). **^13^C-NMR** (100 MHz, CDCl_3_): δ = 172.1 (4C, Ar—**C_q_**), 160.5 (2C, Carbonyl-**C_q_**), 155.8 (2C, Ar—**C_q_**), 148.5 (2C, Ar—**C_q_**), 139.8 (1C, Ar—**C**—H), 126.4 (2C, Ar—**C**—H), 86.0 (2C, Ar—**C**—H), 54.2 (4C, O—**C**H_3_). **IR**: 3460, 3386, 3245, 3101, 3052, 2996, 2924, 2854, 2580, 2163, 1708, 1598, 1573, 1509, 1478, 1455, 1424, 1365, 1300, 1256, 1217, 1190, 1162, 1141, 1094, 1063, 1041, 1000, 987, 958, 909, 879, 856, 834, 777, 754, 696, 663 cm^−1^.

**Tennimide macrocycle [4:4 product or** (**I**)**] ^1^H-NMR** (400 MHz, CDCl_3_): δ = 8.13 (*m*, 2H, Ar—**H**), 8.03 (*m*, 2H, Ar—**H**), 7.96 (*s*, 4H, Ar—**H**), 7.80 (*m*, 2H, Ar—**H**), 7.72 (*m*, 2H, Ar—**H**), 5.67 (*s*, 2H, Ar—**H**), 5.63 (*s*, 2H, Ar—**H**), 3.58 (*s*, 12H, O—C**H_3_**), 3.50 (*s*, 12H, O—C**H_3_**). **IR**: 3100, 2957, 2924, 2853, 1733, 1711, 1586, 1557, 1467, 1402, 1363, 1309, 1282, 1192, 1157, 1086, 1077, 1054, 995, 937, 923, 842, 824, 778, 741, 709, 652 cm^−1^.

## 5. Refinement

Crystal data, data collection and structure refinement details are summarized in Table 2 ▸. H atoms attached to C atoms were treated as riding using the *SHELXL14* (Sheldrick, 2015*b* ▸) defaults at 294 (1) K with C—H = 0.93 Å (aromatic) and *U*_iso_(H) = 1.2*U*_eq_(C) (aromatic): the methyl C—H = 0.96 Å (aliphatic) and *U*_iso_(H) = 1.5*U*_eq_(C). The H atoms of the partial occupancy water mol­ecule were treated using three DFIX restraints at chemically sensible positions and directed towards the closest O=C acceptor groups. The presence of the water in this location is similar to that noted in a 26(BrIO)_4_ structure (XOCHUU; Mocilac & Gallagher, 2014 ▸) where a hemihydrate spans a pyridine N atom and a carbonyl O=C by inter­molecular hydrogen bonding at the macrocycle cavity entrance.

Structural analysis in the penultimate stages of refinement demonstrates that by omitting the partial occupancy water mol­ecule, the *R*-factor increases from 0.054 to 0.056. The residual electron density increases from +0.19 to 0.58 e A^−3^, resulting in a single peak of residual electron density on the twofold axis. This is where the partial occupancy water mol­ecule is located. The WGHT card increases from 0.067 to 0.081. There is no other atom or group disorder in the structure of (**I**).

## Supplementary Material

Crystal structure: contains datablock(s) global, I. DOI: 10.1107/S2056989023006837/ex2073sup1.cif

Structure factors: contains datablock(s) I. DOI: 10.1107/S2056989023006837/ex2073Isup2.hkl

CCDC reference: 2286597

Additional supporting information:  crystallographic information; 3D view; checkCIF report

## Figures and Tables

**Figure 3:**
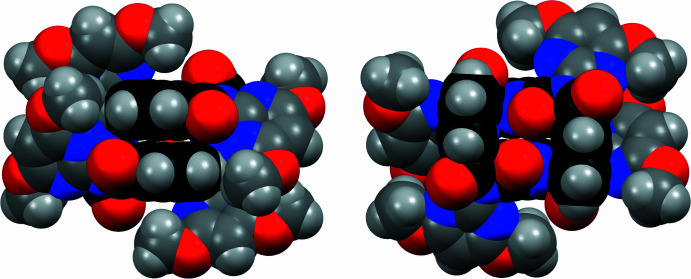
Two views of (**I**) with all atoms drawn as their van der Waals spheres and the central pyridine backbone drawn in black using the *Mercury* program (Macrae *et al.*, 2020 ▸).

**Table 1:** Hydrogen-bond geometry (Å, °)

*D*—H⋯*A*	*D*—H	H⋯*A*	*D*⋯*A*	*D*—H⋯*A*
C15—H15⋯O3^i^	0.93	2.47	3.148 (4)	130
C15—H15⋯O4^ii^	0.93	2.42	3.084 (4)	129
C18*A*—H18*C*⋯O23*A*	0.96	2.47	3.393 (6)	160
C24—H24⋯O1*W* ^iii^	0.93	2.49	3.14 (2)	127
C26—H26⋯N22*A*	0.93	2.62	3.325 (4)	133
C27*A*—H27*B*⋯O23*A* ^iv^	0.96	2.57	3.144 (4)	119
O1*W*—H1*W*⋯O2	0.85	2.32	2.97 (3)	134 (5)

Symmetry codes: (i) 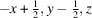 ; (ii) 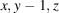 ; (iii) 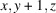 ; (iv) 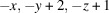 .

**Table 2:** Experimental details

Crystal data	
Chemical formula	C_52_H_40_N_16_O_16_·0.167H_2_O
*M*_r_	1148.02
Crystal system, space group	Orthorhombic, *P* *b* *c* *n*
Temperature (K)	294
*a*, *b*, *c* (Å)	18.8065 (10), 10.0745 (6), 28.847 (3)
*V* (Å^3^)	5465.6 (7)
*Z*	4
Radiation type	Cu *K*α
μ (mm^−1^)	0.91
Crystal size (mm)	0.39 × 0.30 × 0.04

Data collection	
Diffractometer	Xcalibur, Sapphire3, Gemini Ultra
Absorption correction	Analytical (*ABSFAC*; Clark & Reid, 1998 ▸)
*T*_min_, *T*_max_	0.778, 0.973
No. of measured, independent and observed [*I* > 2σ(*I*)] reflections	14675, 4439, 3036
*R*_int_	0.038
(sin θ/λ)_max_ (Å^−1^)	0.581

Refinement	
*R*[*F* ^2^ > 2σ(*F* ^2^)], *wR*(*F* ^2^), *S*	0.054, 0.159, 1.03
No. of reflections	4439
No. of parameters	392
No. of restraints	3
H-atom treatment	H atoms treated by a mixture of independent and constrained refinement
Δρ_max_, Δρ_min_ (e Å^−3^)	0.19, −0.18

Computer programs: *CrysAlis PRO* (Rigaku OD, 2015 ▸), *SHELXT14/6* (Sheldrick, 2015*a* ▸), *SHELXL14/6* (Sheldrick, 2015*b* ▸), *PLATON* (Spek, 2020 ▸) and *Mercury* (Macrae *et al.*, 2020 ▸).

## supplementary crystallographic information

### Crystal data

**Table:**

C_52_H_40_N_16_O_16_·0.167H_2_O	*D*_x_ = 1.395 Mg m^−^^3^
*M**_r_* = 1148.02	Cu *K*α radiation, λ = 1.54184 Å
Orthorhombic, *P**b**c**n*	Cell parameters from 2330 reflections
*a* = 18.8065 (10) Å	θ = 2.8–63.6°
*b* = 10.0745 (6) Å	µ = 0.91 mm^−^^1^
*c* = 28.847 (3) Å	*T* = 294 K
*V* = 5465.6 (7) Å^3^	Plate, colourless
*Z* = 4	0.39 × 0.30 × 0.04 mm
*F*(000) = 2374.7	

### Data collection

**Table:**

Xcalibur, Sapphire3, Gemini Ultra diffractometer	3036 reflections with *I* > 2σ(*I*)
Radiation source: Enhance Ultra (Cu) X-ray Source	*R*_int_ = 0.038
Mirror monochromator	θ_max_ = 63.6°, θ_min_ = 3.1°
ω scans	*h* = −19→21
Absorption correction: analytical (*ABSFAC*; Clark & Reid, 1998)	*k* = −6→11
*T*_min_ = 0.778, *T*_max_ = 0.973	*l* = −33→30
14675 measured reflections	2330 standard reflections every 60 min
4439 independent reflections	intensity decay: 1%

### Refinement

**Table:**

Refinement on *F*^2^	Secondary atom site location: inferred from neighbouring sites
Least-squares matrix: full	Hydrogen site location: mixed
*R*[*F*^2^ > 2σ(*F*^2^)] = 0.054	H atoms treated by a mixture of independent and constrained refinement
*wR*(*F*^2^) = 0.159	*w* = 1/[σ^2^(*F*_o_^2^) + (0.0686*P*)^2^ + 1.3472*P*] where *P* = (*F*_o_^2^ + 2*F*_c_^2^)/3
*S* = 1.03	(Δ/σ)_max_ < 0.001
4439 reflections	Δρ_max_ = 0.19 e Å^−^^3^
392 parameters	Δρ_min_ = −0.18 e Å^−^^3^
3 restraints	Extinction correction: SHELXL, Fc^*^=kFc[1+0.001xFc^2^λ^3^/sin(2θ)]^-1/4^
Primary atom site location: structure-invariant direct methods	Extinction coefficient: 0.00055 (7)

### Special details

**Table:**

Geometry. All esds (except the esd in the dihedral angle between two l.s. planes) are estimated using the full covariance matrix. The cell esds are taken into account individually in the estimation of esds in distances, angles and torsion angles; correlations between esds in cell parameters are only used when they are defined by crystal symmetry. An approximate (isotropic) treatment of cell esds is used for estimating esds involving l.s. planes.

### Fractional atomic coordinates and isotropic or equivalent isotropic displacement parameters (Å^2^)

**Table:**

	*x*	*y*	*z*	*U*_iso_*/*U*_eq_	Occ. (<1)
O1	−0.05721 (12)	0.3212 (2)	0.40663 (8)	0.0750 (6)	
C1	−0.03715 (15)	0.3931 (3)	0.37594 (10)	0.0543 (7)	
N1	−0.07837 (12)	0.4970 (2)	0.35800 (8)	0.0544 (6)	
O2	−0.06477 (11)	0.43970 (19)	0.28185 (7)	0.0598 (5)	
C2	−0.07302 (13)	0.5274 (3)	0.30973 (10)	0.0504 (7)	
O3	0.22174 (11)	0.6319 (2)	0.29594 (7)	0.0652 (6)	
C3	0.16807 (15)	0.6223 (3)	0.31815 (10)	0.0527 (7)	
N3	0.13350 (13)	0.7385 (2)	0.33702 (8)	0.0548 (6)	
O4	0.11289 (15)	0.9585 (2)	0.32596 (8)	0.0876 (8)	
C4	0.11694 (17)	0.8484 (3)	0.31003 (11)	0.0619 (8)	
C11	0.13402 (14)	0.4934 (3)	0.33012 (10)	0.0499 (6)	
N12	0.06713 (11)	0.4984 (2)	0.34536 (7)	0.0487 (5)	
C13	0.03634 (14)	0.3830 (3)	0.35609 (9)	0.0487 (6)	
C14	0.07002 (16)	0.2620 (3)	0.35217 (11)	0.0621 (8)	
H14	0.0469	0.1839	0.3605	0.074*	
C15	0.13824 (17)	0.2597 (3)	0.33575 (13)	0.0738 (10)	
H15	0.1622	0.1794	0.3324	0.089*	
C16	0.17103 (16)	0.3770 (3)	0.32431 (12)	0.0655 (8)	
H16	0.2173	0.3776	0.3129	0.079*	
C11A	−0.13160 (16)	0.5612 (3)	0.38435 (10)	0.0612 (8)	
N12A	−0.18786 (13)	0.5977 (3)	0.36019 (9)	0.0684 (7)	
C13A	−0.2347 (2)	0.6711 (4)	0.38399 (14)	0.0897 (12)	
C14A	−0.2263 (2)	0.6991 (5)	0.43042 (14)	0.1027 (14)	
H14A	−0.2600	0.7475	0.4469	0.123*	
C15A	−0.1659 (2)	0.6523 (4)	0.45107 (12)	0.0858 (11)	
N16A	−0.11615 (15)	0.5832 (3)	0.42871 (9)	0.0714 (8)	
O13A	−0.29127 (15)	0.7190 (4)	0.36166 (12)	0.1228 (11)	
C17A	−0.2926 (3)	0.7082 (6)	0.31264 (19)	0.1275 (18)	
H17A	−0.2552	0.7613	0.2996	0.191*	
H17B	−0.3377	0.7387	0.3012	0.191*	
H17C	−0.2858	0.6171	0.3039	0.191*	
O15A	−0.15514 (19)	0.6812 (3)	0.49625 (9)	0.1107 (10)	
C18A	−0.0908 (3)	0.6365 (5)	0.51825 (14)	0.1134 (15)	
H18A	−0.0829	0.5449	0.5108	0.170*	
H18B	−0.0952	0.6462	0.5512	0.170*	
H18C	−0.0515	0.6887	0.5074	0.170*	
C21	−0.07603 (14)	0.6707 (3)	0.29661 (9)	0.0504 (7)	
N22	−0.09433 (12)	0.6954 (2)	0.25293 (8)	0.0502 (6)	
C23	−0.09991 (17)	0.8212 (3)	0.23984 (10)	0.0581 (7)	
C24	−0.0874 (2)	0.9276 (3)	0.26911 (13)	0.0771 (10)	
H24	−0.0937	1.0146	0.2591	0.093*	
C25	−0.0652 (2)	0.9002 (3)	0.31365 (13)	0.0816 (11)	
H25	−0.0547	0.9689	0.3341	0.098*	
C26	−0.05880 (17)	0.7711 (3)	0.32738 (11)	0.0670 (8)	
H26	−0.0430	0.7508	0.3571	0.080*	
C21A	0.12724 (17)	0.7424 (3)	0.38590 (10)	0.0580 (8)	
N22A	0.07257 (14)	0.8089 (2)	0.40337 (9)	0.0644 (7)	
C23A	0.06838 (19)	0.8052 (3)	0.44966 (11)	0.0704 (9)	
C24A	0.1147 (2)	0.7351 (3)	0.47695 (12)	0.0773 (10)	
H24A	0.1097	0.7312	0.5090	0.093*	
C25A	0.1687 (2)	0.6709 (3)	0.45415 (12)	0.0705 (9)	
N26A	0.17692 (14)	0.6744 (2)	0.40795 (9)	0.0630 (7)	
O23A	0.01530 (15)	0.8719 (2)	0.47037 (8)	0.0903 (8)	
C27A	−0.0336 (2)	0.9460 (4)	0.44195 (14)	0.1050 (14)	
H27A	−0.0076	1.0071	0.4228	0.158*	
H27B	−0.0659	0.9944	0.4614	0.158*	
H27C	−0.0599	0.8860	0.4226	0.158*	
O25A	0.21549 (15)	0.6015 (3)	0.47933 (8)	0.0899 (8)	
C28A	0.2726 (2)	0.5372 (5)	0.45500 (16)	0.1113 (15)	
H28A	0.2535	0.4717	0.4343	0.167*	
H28B	0.3037	0.4950	0.4769	0.167*	
H28C	0.2988	0.6019	0.4376	0.167*	
O1W	0.0000	0.187 (3)	0.2500	0.180 (15)	0.167
H1W	−0.0358 (4)	0.238 (3)	0.248 (3)	0.100*	0.167

### Atomic displacement parameters (Å^2^)

**Table:**

	*U*^11^	*U*^22^	*U*^33^	*U*^12^	*U*^13^	*U*^23^
O1	0.0701 (14)	0.0827 (15)	0.0721 (14)	−0.0008 (12)	0.0112 (12)	0.0203 (12)
C1	0.0507 (17)	0.0590 (17)	0.0533 (17)	−0.0002 (14)	−0.0006 (13)	−0.0020 (14)
N1	0.0467 (13)	0.0683 (15)	0.0483 (13)	0.0126 (12)	0.0028 (10)	0.0008 (11)
O2	0.0654 (13)	0.0597 (12)	0.0544 (12)	0.0076 (10)	−0.0001 (10)	−0.0033 (10)
C2	0.0391 (15)	0.0606 (17)	0.0515 (16)	0.0028 (13)	−0.0011 (12)	−0.0036 (14)
O3	0.0487 (12)	0.0717 (14)	0.0752 (14)	−0.0099 (10)	−0.0037 (10)	−0.0028 (10)
C3	0.0463 (16)	0.0493 (16)	0.0625 (18)	−0.0019 (13)	−0.0076 (14)	−0.0046 (13)
N3	0.0650 (15)	0.0426 (12)	0.0567 (14)	−0.0054 (11)	−0.0084 (12)	−0.0050 (10)
O4	0.129 (2)	0.0410 (12)	0.0928 (17)	−0.0117 (13)	−0.0023 (15)	−0.0104 (11)
C4	0.072 (2)	0.0402 (16)	0.073 (2)	−0.0086 (14)	0.0004 (16)	−0.0058 (14)
C11	0.0437 (15)	0.0464 (15)	0.0596 (16)	−0.0008 (13)	−0.0039 (13)	−0.0072 (12)
N12	0.0469 (13)	0.0459 (12)	0.0532 (13)	0.0028 (11)	−0.0017 (10)	−0.0055 (10)
C13	0.0459 (15)	0.0459 (15)	0.0541 (16)	−0.0008 (12)	−0.0036 (12)	0.0004 (12)
C14	0.0559 (18)	0.0419 (15)	0.088 (2)	−0.0023 (14)	0.0000 (16)	−0.0003 (14)
C15	0.0575 (19)	0.0411 (16)	0.123 (3)	0.0085 (15)	0.0102 (19)	−0.0078 (17)
C16	0.0487 (17)	0.0519 (17)	0.096 (2)	0.0026 (14)	0.0069 (16)	−0.0086 (15)
C11A	0.0531 (18)	0.072 (2)	0.0581 (19)	0.0104 (16)	0.0079 (14)	−0.0033 (15)
N12A	0.0498 (15)	0.0873 (19)	0.0681 (17)	0.0140 (14)	0.0091 (12)	−0.0036 (14)
C13A	0.068 (2)	0.110 (3)	0.091 (3)	0.029 (2)	0.015 (2)	−0.007 (2)
C14A	0.095 (3)	0.133 (4)	0.080 (3)	0.038 (3)	0.025 (2)	−0.019 (2)
C15A	0.101 (3)	0.095 (3)	0.061 (2)	0.016 (2)	0.015 (2)	−0.0103 (19)
N16A	0.0756 (18)	0.0832 (19)	0.0553 (16)	0.0099 (15)	0.0056 (13)	−0.0074 (13)
O13A	0.0773 (19)	0.177 (3)	0.114 (2)	0.062 (2)	0.0076 (17)	−0.011 (2)
C17A	0.092 (3)	0.164 (5)	0.126 (4)	0.048 (3)	−0.036 (3)	−0.026 (4)
O15A	0.147 (3)	0.124 (2)	0.0608 (16)	0.022 (2)	0.0187 (17)	−0.0204 (15)
C18A	0.145 (4)	0.128 (4)	0.067 (3)	0.002 (3)	−0.006 (3)	−0.008 (2)
C21	0.0460 (15)	0.0531 (16)	0.0520 (16)	−0.0005 (13)	0.0010 (12)	−0.0060 (13)
N22	0.0504 (13)	0.0455 (13)	0.0547 (14)	0.0051 (10)	−0.0002 (10)	−0.0015 (10)
C23	0.0652 (19)	0.0432 (16)	0.0660 (19)	0.0025 (14)	0.0029 (15)	−0.0047 (14)
C24	0.097 (3)	0.0496 (18)	0.085 (3)	0.0003 (18)	0.004 (2)	−0.0084 (16)
C25	0.105 (3)	0.064 (2)	0.076 (2)	−0.013 (2)	−0.003 (2)	−0.0195 (18)
C26	0.069 (2)	0.071 (2)	0.0607 (19)	−0.0050 (17)	−0.0029 (16)	−0.0110 (15)
C21A	0.067 (2)	0.0444 (15)	0.0626 (19)	−0.0084 (15)	−0.0097 (16)	−0.0120 (13)
N22A	0.0752 (18)	0.0561 (15)	0.0617 (16)	−0.0021 (13)	−0.0107 (13)	−0.0155 (12)
C23A	0.086 (2)	0.0623 (19)	0.063 (2)	−0.0023 (18)	−0.0076 (18)	−0.0193 (16)
C24A	0.100 (3)	0.074 (2)	0.058 (2)	0.000 (2)	−0.0156 (19)	−0.0111 (17)
C25A	0.082 (2)	0.0599 (19)	0.070 (2)	−0.0039 (17)	−0.0193 (19)	−0.0057 (16)
N26A	0.0690 (17)	0.0546 (14)	0.0654 (16)	−0.0052 (13)	−0.0150 (13)	−0.0086 (12)
O23A	0.110 (2)	0.0919 (17)	0.0689 (15)	0.0239 (16)	−0.0025 (14)	−0.0257 (13)
C27A	0.112 (3)	0.111 (3)	0.091 (3)	0.039 (3)	−0.015 (3)	−0.039 (2)
O25A	0.0992 (19)	0.0954 (18)	0.0752 (16)	0.0111 (15)	−0.0286 (15)	−0.0032 (13)
C28A	0.103 (3)	0.121 (4)	0.110 (3)	0.033 (3)	−0.026 (3)	−0.004 (3)
O1W	0.17 (3)	0.085 (19)	0.28 (4)	0.000	−0.06 (3)	0.000

### Geometric parameters (Å, º)

**Table:**

O1—C1	1.205 (3)	C17A—H17B	0.9600
C1—N1	1.402 (3)	C17A—H17C	0.9600
C1—C13	1.499 (4)	O15A—C18A	1.438 (5)
N1—C11A	1.414 (3)	C18A—H18A	0.9600
N1—C2	1.429 (3)	C18A—H18B	0.9600
O2—C2	1.205 (3)	C18A—H18C	0.9600
C2—C21	1.494 (4)	C21—N22	1.330 (3)
O3—C3	1.200 (3)	C21—C26	1.384 (4)
C3—N3	1.446 (4)	N22—C23	1.327 (3)
C3—C11	1.488 (4)	C23—C24	1.385 (4)
N3—C4	1.389 (4)	C23—C4^i^	1.499 (4)
N3—C21A	1.416 (4)	C24—C25	1.379 (5)
O4—C4	1.203 (3)	C24—H24	0.9300
C4—C23^i^	1.499 (4)	C25—C26	1.365 (5)
C11—N12	1.333 (3)	C25—H25	0.9300
C11—C16	1.374 (4)	C26—H26	0.9300
N12—C13	1.335 (3)	C21A—N26A	1.322 (4)
C13—C14	1.378 (4)	C21A—N22A	1.326 (4)
C14—C15	1.368 (4)	N22A—C23A	1.338 (4)
C14—H14	0.9300	C23A—O23A	1.343 (4)
C15—C16	1.373 (4)	C23A—C24A	1.370 (5)
C15—H15	0.9300	C24A—C25A	1.373 (5)
C16—H16	0.9300	C24A—H24A	0.9300
C11A—N12A	1.319 (4)	C25A—O25A	1.338 (4)
C11A—N16A	1.331 (4)	C25A—N26A	1.342 (4)
N12A—C13A	1.339 (4)	O23A—C27A	1.441 (4)
C13A—O13A	1.334 (4)	C27A—H27A	0.9600
C13A—C14A	1.378 (5)	C27A—H27B	0.9600
C14A—C15A	1.367 (5)	C27A—H27C	0.9600
C14A—H14A	0.9300	O25A—C28A	1.438 (5)
C15A—N16A	1.332 (4)	C28A—H28A	0.9600
C15A—O15A	1.351 (4)	C28A—H28B	0.9600
O13A—C17A	1.418 (5)	C28A—H28C	0.9600
C17A—H17A	0.9600	O1W—H1W	0.850 (5)
			
O1—C1—N1	123.2 (3)	H17A—C17A—H17C	109.5
O1—C1—C13	121.9 (3)	H17B—C17A—H17C	109.5
N1—C1—C13	114.8 (2)	C15A—O15A—C18A	119.0 (3)
C1—N1—C11A	122.3 (2)	O15A—C18A—H18A	109.5
C1—N1—C2	118.7 (2)	O15A—C18A—H18B	109.5
C11A—N1—C2	118.4 (2)	H18A—C18A—H18B	109.5
O2—C2—N1	120.1 (3)	O15A—C18A—H18C	109.5
O2—C2—C21	123.0 (3)	H18A—C18A—H18C	109.5
N1—C2—C21	116.8 (2)	H18B—C18A—H18C	109.5
O3—C3—N3	120.9 (3)	N22—C21—C26	122.1 (3)
O3—C3—C11	123.8 (3)	N22—C21—C2	115.5 (2)
N3—C3—C11	115.2 (3)	C26—C21—C2	122.3 (3)
C4—N3—C21A	121.2 (2)	C23—N22—C21	117.9 (2)
C4—N3—C3	122.4 (2)	N22—C23—C24	123.6 (3)
C21A—N3—C3	115.8 (2)	N22—C23—C4^i^	117.7 (2)
O4—C4—N3	122.3 (3)	C24—C23—C4^i^	118.7 (3)
O4—C4—C23^i^	121.4 (3)	C25—C24—C23	117.7 (3)
N3—C4—C23^i^	116.1 (2)	C25—C24—H24	121.2
N12—C11—C16	123.4 (3)	C23—C24—H24	121.2
N12—C11—C3	116.7 (2)	C26—C25—C24	119.2 (3)
C16—C11—C3	119.9 (3)	C26—C25—H25	120.4
C11—N12—C13	116.9 (2)	C24—C25—H25	120.4
N12—C13—C14	123.5 (3)	C25—C26—C21	119.3 (3)
N12—C13—C1	115.4 (2)	C25—C26—H26	120.3
C14—C13—C1	121.0 (3)	C21—C26—H26	120.3
C15—C14—C13	118.4 (3)	N26A—C21A—N22A	128.8 (3)
C15—C14—H14	120.8	N26A—C21A—N3	114.0 (3)
C13—C14—H14	120.8	N22A—C21A—N3	117.2 (3)
C14—C15—C16	119.3 (3)	C21A—N22A—C23A	114.3 (3)
C14—C15—H15	120.4	N22A—C23A—O23A	118.3 (3)
C16—C15—H15	120.4	N22A—C23A—C24A	123.4 (3)
C15—C16—C11	118.5 (3)	O23A—C23A—C24A	118.3 (3)
C15—C16—H16	120.7	C23A—C24A—C25A	116.0 (3)
C11—C16—H16	120.7	C23A—C24A—H24A	122.0
N12A—C11A—N16A	129.5 (3)	C25A—C24A—H24A	122.0
N12A—C11A—N1	114.3 (3)	O25A—C25A—N26A	118.5 (3)
N16A—C11A—N1	116.0 (3)	O25A—C25A—C24A	118.2 (3)
C11A—N12A—C13A	114.2 (3)	N26A—C25A—C24A	123.3 (3)
O13A—C13A—N12A	118.5 (4)	C21A—N26A—C25A	114.2 (3)
O13A—C13A—C14A	119.1 (3)	C23A—O23A—C27A	118.7 (3)
N12A—C13A—C14A	122.4 (4)	O23A—C27A—H27A	109.5
C15A—C14A—C13A	116.6 (3)	O23A—C27A—H27B	109.5
C15A—C14A—H14A	121.7	H27A—C27A—H27B	109.5
C13A—C14A—H14A	121.7	O23A—C27A—H27C	109.5
N16A—C15A—O15A	118.3 (4)	H27A—C27A—H27C	109.5
N16A—C15A—C14A	123.6 (3)	H27B—C27A—H27C	109.5
O15A—C15A—C14A	118.1 (3)	C25A—O25A—C28A	117.5 (3)
C11A—N16A—C15A	113.5 (3)	O25A—C28A—H28A	109.5
C13A—O13A—C17A	117.9 (3)	O25A—C28A—H28B	109.5
O13A—C17A—H17A	109.5	H28A—C28A—H28B	109.5
O13A—C17A—H17B	109.5	O25A—C28A—H28C	109.5
H17A—C17A—H17B	109.5	H28A—C28A—H28C	109.5
O13A—C17A—H17C	109.5	H28B—C28A—H28C	109.5
			
O1—C1—N1—C11A	26.2 (4)	C13A—C14A—C15A—N16A	0.4 (7)
C13—C1—N1—C11A	−150.1 (3)	C13A—C14A—C15A—O15A	178.3 (4)
O1—C1—N1—C2	−145.4 (3)	N12A—C11A—N16A—C15A	0.1 (5)
C13—C1—N1—C2	38.4 (3)	N1—C11A—N16A—C15A	175.5 (3)
C1—N1—C2—O2	35.9 (4)	O15A—C15A—N16A—C11A	−179.4 (3)
C11A—N1—C2—O2	−136.0 (3)	C14A—C15A—N16A—C11A	−1.5 (6)
C1—N1—C2—C21	−142.4 (3)	N12A—C13A—O13A—C17A	−10.2 (6)
C11A—N1—C2—C21	45.7 (3)	C14A—C13A—O13A—C17A	169.5 (5)
O3—C3—N3—C4	51.6 (4)	N16A—C15A—O15A—C18A	0.0 (6)
C11—C3—N3—C4	−131.0 (3)	C14A—C15A—O15A—C18A	−178.0 (4)
O3—C3—N3—C21A	−119.2 (3)	O2—C2—C21—N22	23.0 (4)
C11—C3—N3—C21A	58.3 (3)	N1—C2—C21—N22	−158.8 (2)
C21A—N3—C4—O4	17.8 (5)	O2—C2—C21—C26	−155.0 (3)
C3—N3—C4—O4	−152.5 (3)	N1—C2—C21—C26	23.2 (4)
C21A—N3—C4—C23^i^	−158.6 (3)	C26—C21—N22—C23	−3.9 (4)
C3—N3—C4—C23^i^	31.1 (4)	C2—C21—N22—C23	178.0 (2)
O3—C3—C11—N12	−165.0 (3)	C21—N22—C23—C24	0.3 (5)
N3—C3—C11—N12	17.6 (4)	C21—N22—C23—C4^i^	177.0 (3)
O3—C3—C11—C16	13.7 (4)	N22—C23—C24—C25	2.7 (5)
N3—C3—C11—C16	−163.7 (3)	C4^i^—C23—C24—C25	−174.0 (3)
C16—C11—N12—C13	1.3 (4)	C23—C24—C25—C26	−2.1 (6)
C3—C11—N12—C13	179.9 (2)	C24—C25—C26—C21	−1.3 (5)
C11—N12—C13—C14	0.0 (4)	N22—C21—C26—C25	4.5 (5)
C11—N12—C13—C1	176.3 (2)	C2—C21—C26—C25	−177.6 (3)
O1—C1—C13—N12	−141.8 (3)	C4—N3—C21A—N26A	−143.9 (3)
N1—C1—C13—N12	34.4 (3)	C3—N3—C21A—N26A	27.0 (3)
O1—C1—C13—C14	34.5 (4)	C4—N3—C21A—N22A	38.0 (4)
N1—C1—C13—C14	−149.2 (3)	C3—N3—C21A—N22A	−151.2 (2)
N12—C13—C14—C15	−1.1 (5)	N26A—C21A—N22A—C23A	0.1 (4)
C1—C13—C14—C15	−177.1 (3)	N3—C21A—N22A—C23A	178.0 (3)
C13—C14—C15—C16	0.8 (5)	C21A—N22A—C23A—O23A	178.8 (3)
C14—C15—C16—C11	0.4 (5)	C21A—N22A—C23A—C24A	−2.4 (5)
N12—C11—C16—C15	−1.5 (5)	N22A—C23A—C24A—C25A	2.4 (5)
C3—C11—C16—C15	179.9 (3)	O23A—C23A—C24A—C25A	−178.8 (3)
C1—N1—C11A—N12A	−144.5 (3)	C23A—C24A—C25A—O25A	179.7 (3)
C2—N1—C11A—N12A	27.1 (4)	C23A—C24A—C25A—N26A	−0.3 (5)
C1—N1—C11A—N16A	39.5 (4)	N22A—C21A—N26A—C25A	1.8 (4)
C2—N1—C11A—N16A	−148.9 (3)	N3—C21A—N26A—C25A	−176.1 (2)
N16A—C11A—N12A—C13A	2.3 (5)	O25A—C25A—N26A—C21A	178.3 (3)
N1—C11A—N12A—C13A	−173.1 (3)	C24A—C25A—N26A—C21A	−1.7 (5)
C11A—N12A—C13A—O13A	176.2 (4)	N22A—C23A—O23A—C27A	−1.2 (5)
C11A—N12A—C13A—C14A	−3.5 (6)	C24A—C23A—O23A—C27A	179.9 (3)
O13A—C13A—C14A—C15A	−177.3 (4)	N26A—C25A—O25A—C28A	1.3 (5)
N12A—C13A—C14A—C15A	2.3 (7)	C24A—C25A—O25A—C28A	−178.7 (3)

Symmetry code: (i) −*x*, *y*, −*z*+1/2.

### Hydrogen-bond geometry (Å, º)

**Table:**

*D*—H···*A*	*D*—H	H···*A*	*D*···*A*	*D*—H···*A*
C15—H15···O3^ii^	0.93	2.47	3.148 (4)	130
C15—H15···O4^iii^	0.93	2.42	3.084 (4)	129
C18*A*—H18*C*···O23*A*	0.96	2.47	3.393 (6)	160
C24—H24···O1*W*^iv^	0.93	2.49	3.14 (2)	127
C26—H26···N22*A*	0.93	2.62	3.325 (4)	133
C27*A*—H27*B*···O23*A*^v^	0.96	2.57	3.144 (4)	119

Symmetry codes: (ii) −*x*+1/2, *y*−1/2, *z*; (iii) *x*, *y*−1, *z*; (iv) *x*, *y*+1, *z*; (v) −*x*, −*y*+2, −*z*+1.
